# Quantifying measurement uncertainties in diode-based in-vivo dosimetry for Cobalt-60 high dose rate brachytherapy

**DOI:** 10.1007/s00411-026-01229-4

**Published:** 2026-06-16

**Authors:** Dilson Lobo, Johan Sunny, M. S. Pooja, Challapalli Srinivas, M. S. Athiyamaan, Sourjya Banerjee, Abhishek Krishna, Paul Simon

**Affiliations:** Department of Radiation Oncology, Kasturba Medical College Mangalore, Manipal Academy of Higher Education, Manipal, India

**Keywords:** In-vivo dosimetry, Diode detector, Measurement uncertainty, Cobalt-60, HDR brachytherapy, Quality assurance, Radiation dosimetry, Dose accuracy, SDG 3: Good Health and Well-being, SDG 9: Industry, Innovation, and Infrastructure

## Abstract

High-dose-rate (HDR) brachytherapy provides a highly conformal cancer treatment modality by exploiting steep dose gradients, and achieving excellent tumour control while minimising radiation exposure to healthy tissues. In-vivo dosimetry (IVD) serves as an essential quality assurance tool, offering independent verification of delivered dose. However, its accuracy can be affected by several measurement-related uncertainties. This study aimed to characterise diode-based IVD for Co-60 HDR brachytherapy and quantify the uncertainties influencing detector performance. A Co-60 HDR afterloading system (SagiNova^®^) was used along with a diode-based in-vivo detector. Calibration was performed using a Polymethyl Methacrylate PMMA phantom. A custom-designed acrylic phantom was fabricated to ensure reproducible detector positioning and fixed geometry during irradiation. Detector linearity and uniformity were assessed by delivering known doses from 1 to 8 Gy in 1 Gy increments. Since conventional brachytherapy treatment planning systems do not account for tissue heterogeneity, additional measurements were performed by placing materials simulating bone (Teflon), lung (cork), and soft tissue (acrylic) of 1–3 cm thickness between the source and detector. The diode exhibited excellent stability, with repeatability showing < 2% relative standard deviation. Sensitivity across cumulative absorbed doses demonstrated < 2.5% variation, confirming strong consistency. A linear response was observed throughout the tested dose range. Heterogeneity analysis revealed notable dose perturbations: as expected, bone-equivalent material produced the highest attenuation, while lung-equivalent material resulted in the least, underscoring the importance of accounting for tissue density variations in IVD measurements. Although IVD offers valuable real-time dose verification in HDR brachytherapy, its widespread clinical adoption remains limited by challenges such as detector size and the steep dose gradients surrounding the source. Comprehensive commissioning—including evaluation of linearity, reproducibility, geometric dependence, and heterogeneity effects—is critical for understanding detector behaviour under clinical conditions. It is concluded that accurate characterisation of these uncertainties enhances the reliability of diode-based IVD systems and supports their integration into routine brachytherapy practice for improved patient safety and treatment precision.

## Introduction

Brachytherapy allows highly localized radiation dose delivery to the target volume in oncologic care due to its ability to deposit very high doses directly into the tumour while achieving rapid dose falloff in adjacent healthy tissues. This steep dose gradient is responsible for excellent tumour control rates, but it also amplifies the need for accurate, real-time verification of the delivered dose. Even minor deviations in source positioning, tissue composition, or applicator geometry can lead to clinically meaningful variations in organ-at-risk (OAR) dose particularly in high-dose-rate (HDR) gynecological brachytherapy, where the rectum and bladder are located mere millimeters away from the radioactive source positions.

Therefore, in vivo dosimetry (IVD) plays a central role in modern brachytherapy quality assurance. By providing independent, real-time verification of the dose actually delivered during treatment, IVD offers an additional layer of patient safety beyond traditional pretreatment verification. It can identify potential errors such as applicator displacement, incorrect source step size, tissue heterogeneities, packing inconsistencies, and anatomical variations between fractions many of which remain undetected by the treatment planning system (TPS) alone.

One major limitation in current TPS-based dose estimation is the assumption of homogeneous tissue composition, which does not reflect real clinical scenarios. Variations in rectal gas content, bony structures, or low-density tissues can significantly affect dose distribution. Kwan et al. (2009) showed that the presence of air in the rectum led to a 14.7% reduction in the actual dose using an Ir-192 source to the anterior rectal wall compared to TPS predictions. This is a clinically significant discrepancy that underscores the necessity for IVD.

Despite its recognized benefits, widespread clinical adoption of IVD has been slow. This is largely due to technological constraints, especially the challenge of developing detectors small enough to function accurately within steep brachytherapy dose gradients. Detectors must be dimensionally compatible with narrow applicator channels, maintain water equivalence, exhibit minimal energy and angular dependence, and remain stable under high-gradient radiation fields (Carrara et al. 2018), while clinical factors such as applicator positioning and rectal or bladder filling can further influence in-vivo detector response (Bergau et al. 2019).

Emerging detectors such as plastic scintillation detectors (PSDs), micro Metal Oxide Semiconductor Field Effect Transistors (MOSFETs), and inorganic scintillator detectors have shown promise. PSDs, in particular, offer a unique combination of real-time readout, excellent spatial resolution, radiation resistance, and near water-equivalence making them highly suitable for HDR applications (Therriault-Proulx et., 2011 & Beddar et al. 2005). Nevertheless, diode detectors remain one of the most practical and commercially accessible options due to their robustness, availability, and compatibility with existing after loader infrastructure.

In vivo dosimetry (IVD) has emerged as an essential component of brachytherapy quality assurance, providing direct verification of the dose delivered under real clinical conditions. However, the steep dose gradients, high dose rates, and complex geometries characteristic of brachytherapy make accurate in vivo measurements challenging. Tanderup et al. highlighted the fundamental limitations and practical considerations of IVD in brachytherapy, emphasizing the need for high-resolution detectors capable of functioning reliably in high-gradient regions (Tanderup et al. 2013). Similarly, Kertzscher et al. (2014) reviewed trends in brachytherapy IVD and underlined the importance of real-time dosimetry for error detection, especially during high-dose-rate (HDR) treatments.

Beyond detector characteristics, the accuracy of IVD is also affected by tissue heterogeneities and non-water materials within the patient or applicator. Traditional TG-43-based dose calculations assume a homogeneous water medium, which may not accurately represent clinical conditions (Mozaffari and Ghorbani 2019). Faucher et al. (2025) further quantified dose perturbations from heterogeneities, emphasizing the need for correction strategies or model-based dose calculation algorithms that incorporate patient-specific anatomy.

Collectively, these studies underscore two key requirements for modern brachytherapy dosimetry: (1) well-characterized, high-resolution detectors capable of providing accurate point-dose measurements under steep gradients; and (2) dosimetric approaches that account for tissue and material heterogeneities to avoid systematic deviations.

## Materials and methods

### Brachytherapy unit and detector

A BEBIG Co-60 remote after loading HDR brachytherapy unit (model Co0.A050; Eckert & Ziegler BEBIG GmbH, Germany) was utilized for this study. The unit supports complex treatments with up to 50 channels and incorporates an integrated IVD module. The air-kerma strength of the Co-60 SagiNova^®^ HDR brachytherapy source was determined according to the TG-43 formalism developed by the American Association of Physicists in Medicine (AAPM), using an air-kerma rate constant of 0.306 mGy·m²·h⁻¹·GBq⁻¹ and an apparent source activity of 7.35 × 10^10^ Bq at the time of measurement (Mozaffari et al., 2019). Source strength was measured using a calibrated cylindrical ionization chamber under reference conditions and independently verified through routine quality assurance and treatment planning system commissioning. This IVD module enables real-time, independent monitoring of rectal and bladder doses directly at the SagiNova^®^ control console, with user-defined dose limits and automatic alerts when thresholds are exceeded. All IVD data are directly incorporated into the treatment report, allowing efficient documentation without the need for additional hardware or interfaces. Two PTW diode detectors (PTW, Freiburg, Germany) were employed: the PTW 9112, a 7-mm flexible probe containing five semiconductor diodes for rectal dose measurement, and the PTW 9111, a 2-mm probe housing a single semiconductor diode designed for bladder dose monitoring during brachytherapy. This study aligns primarily with SDG 3 (Good Health and Well-being) by enhancing the safety and effectiveness of radiotherapy treatments, and secondarily with SDG 9 (Innovation in healthcare technology) through improvements in dosimetric measurement techniques. 

### Calibration of diode detector

**Fig. 1:**
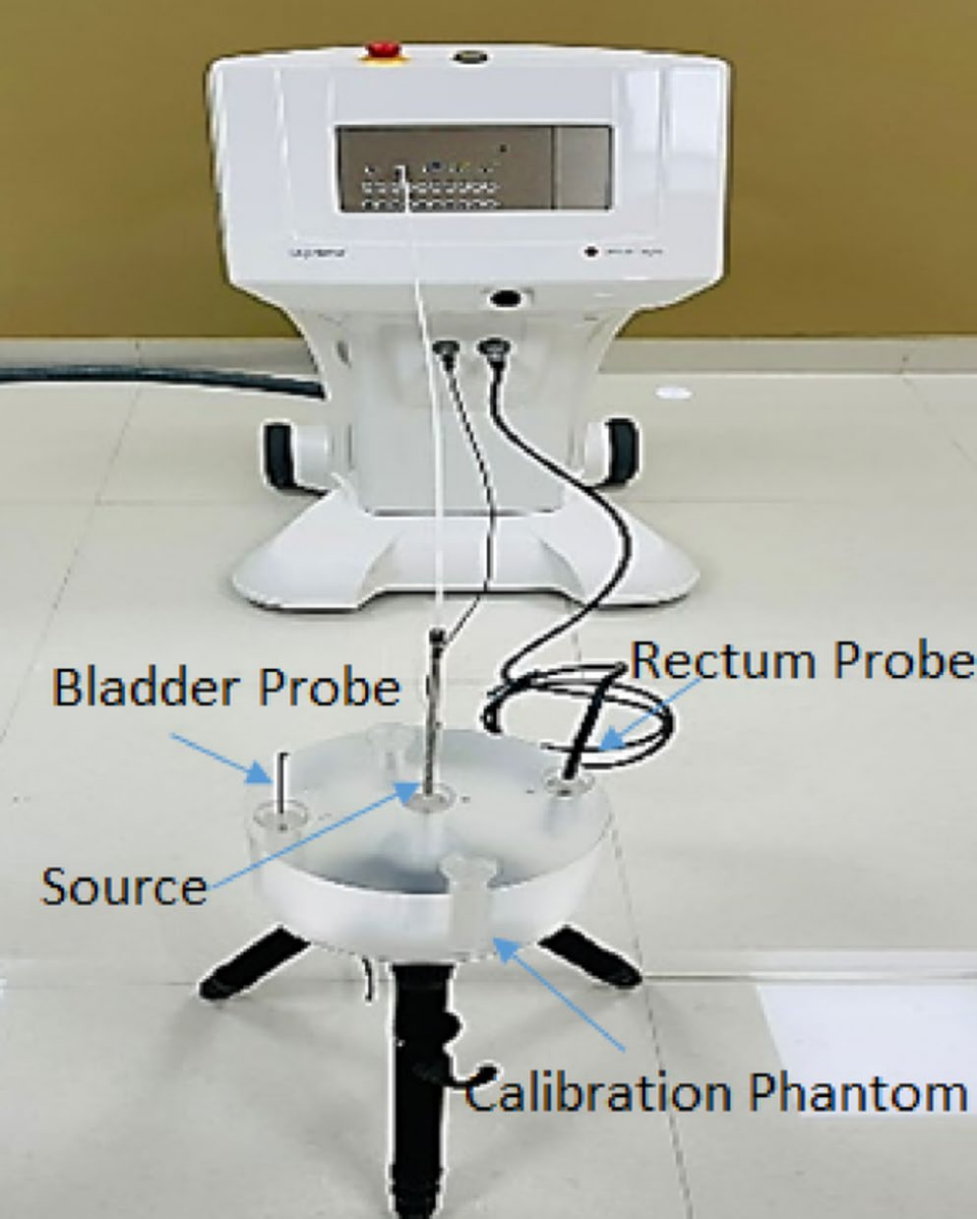
Calibration Phantom used in the present study

The probes were interfaced with the integrated PTW Vivo Dose electrometer embedded within the after loader and operated through the SagiNova^®^ control system. This advanced IVD platform transforms dose verification by enabling simultaneous, real-time monitoring of rectal and bladder doses as the treatment unfolds providing the clinician with immediate feedback and automatic data capture for accurate documentation. The PTW 9112 rectal diode detector was calibrated using a polymethyl methacrylate (PMMA) cylindrical afterloading phantom (Type 9193, PTW, Freiburg, Germany), commonly known as the Krieger phantom, following the manufacturer’s recommended procedure for the SagiNova^®^ Co-60 HDR brachytherapy system (Johan et al 2023). The phantom (20 cm in diameter and 12 cm in height) was mounted on a tripod to reduce backscatter. It included four peripheral channels positioned 8 cm from the central axis. During calibration, the rectal diode detector and source applicator were placed in the phantom as shown in Fig. 1. Each diode (R1–R5) was irradiated for a predefined dwell time, and the collected charge was recorded. Diode-specific calibration factors were then derived using the Treatment Control Computer (TCC) control software and subsequently applied for real-time absorbed dose display during in-vivo measurements.

### Design and fabrication of the phantom for uncertainty measurements

**Fig. 2:**
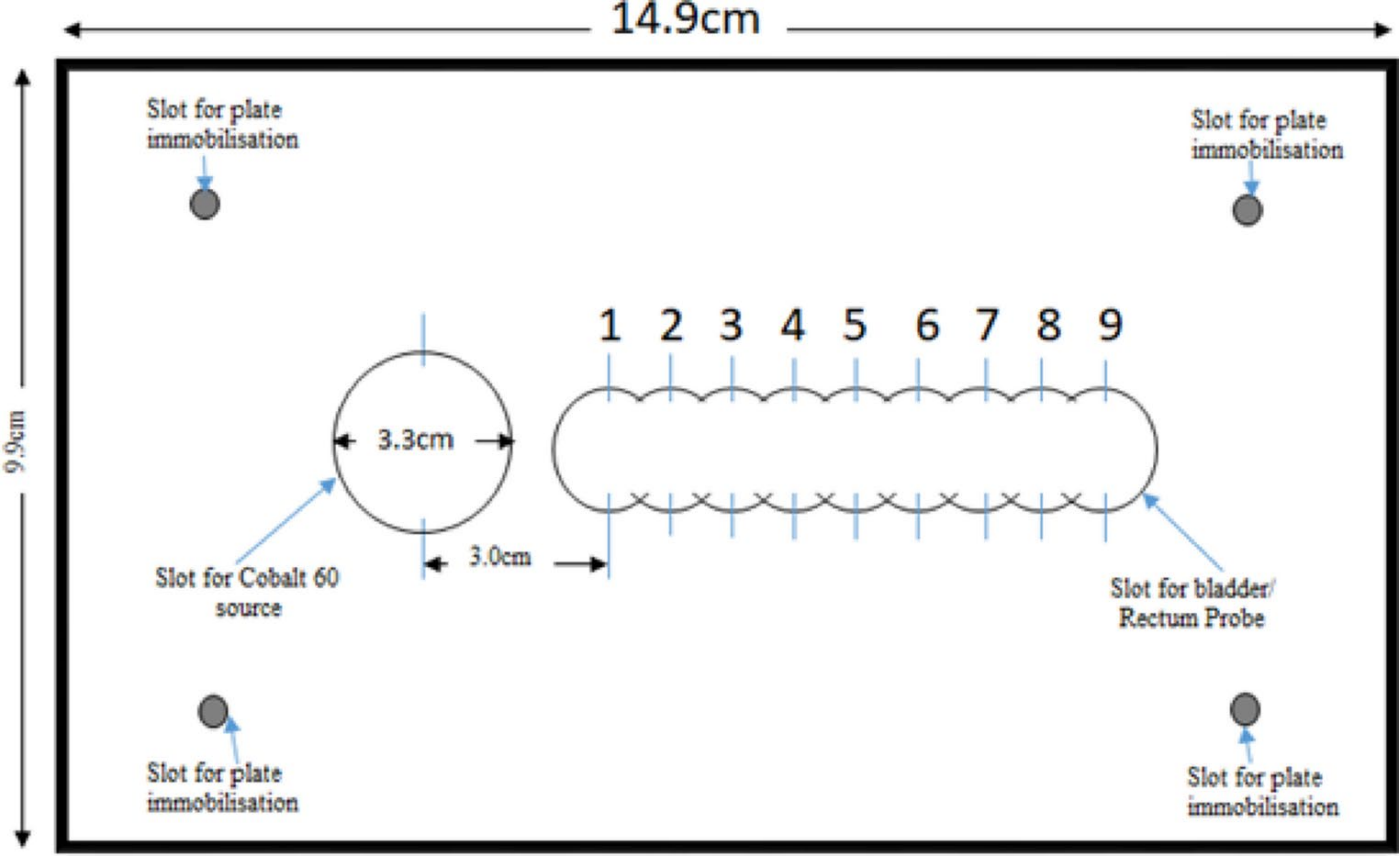
Phantom plate with the slots for placement of detector adopter plugs and adopter for ^60^Co source

To systematically evaluate the measurement uncertainties associated with the use of the diode detector, a customised phantom was designed and fabricated using high-grade acrylic material. The phantom incorporated precisely machined slots to accommodate both the detector adapter plugs and the source transfer adapter, ensuring stable and reproducible positioning during all measurements. Nine detector positions were engineered at predetermined distances from the central source channel, enabling controlled assessment of detector response across varying source–detector distances. This arrangement allowed for a detailed evaluation of positional dependence, dose falloff behaviour, and geometric uncertainties. Figure 2 illustrates the schematic design of the phantom plate, highlighting the nine dedicated slots for detector placement, the Co-60 source channel, and fixtures for immobilizing the plate to maintain consistent geometry throughout the experiments.

**Fig. 3:**
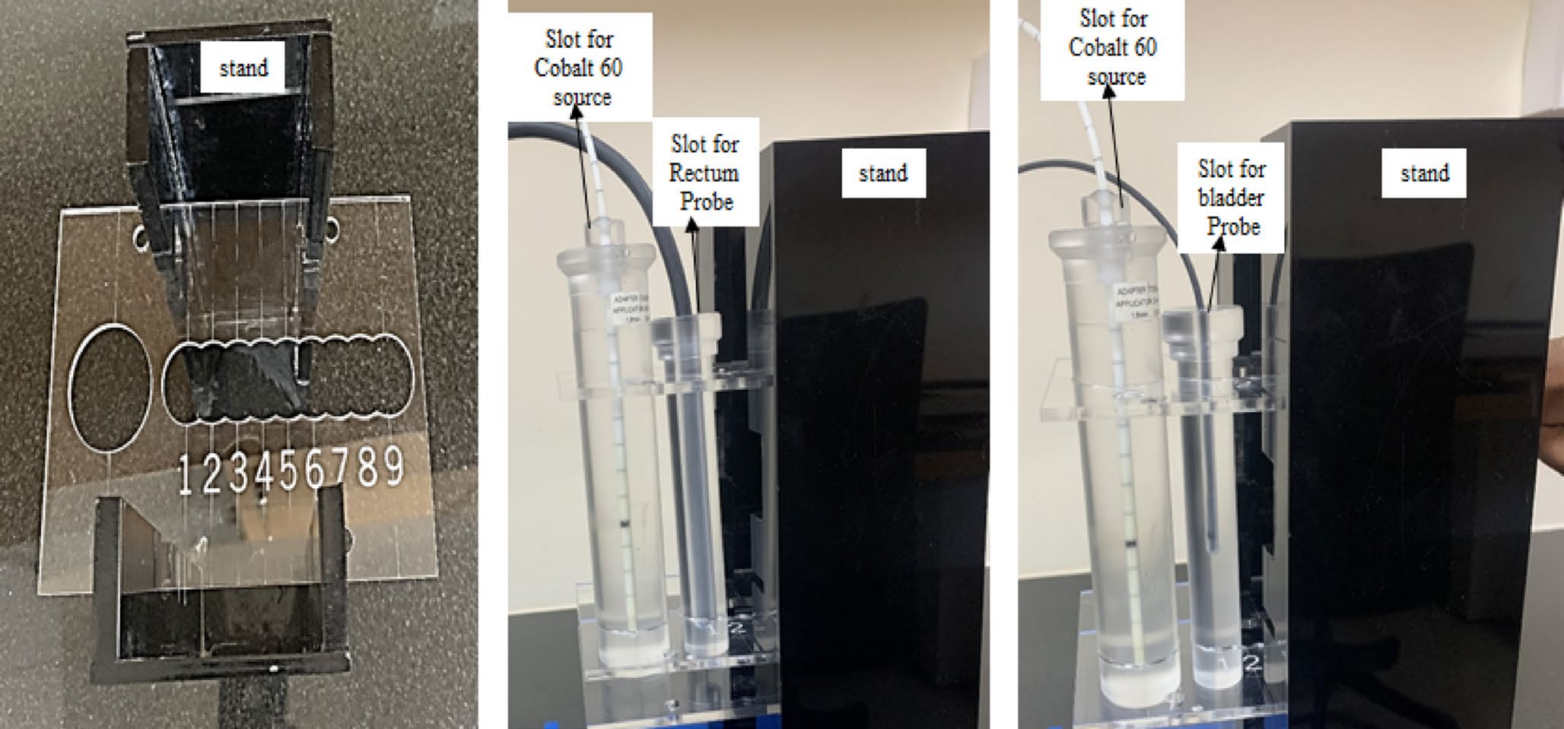
(**a**) Phantom plate showing the slots for placement of detector adaptor plugs and the source transfer adaptor; (**b**) Phantom assembled with the rectal detector and source adaptor plugs in position. (**c**) Phantom assembled with the bladder detector and source adaptor plugs in position

After fabrication, the detector holder was positioned inside a water phantom to simulate full-scatter conditions. The rectal or bladder diode detector and the corresponding dummy source were inserted into their designated slots, ensuring precise and reproducible geometry for all measurements (Fig. 3). An entire setup CT acquisition with 2.5-mm slice thickness was acquired with Wipro GE “High Speed” with a slice thickness of 2.5 mm.

The acquired CT dataset was then imported into the SagiPlan TPS, where the source positions were reconstructed and the diode detector locations were accurately identified. Points of interest were assigned at the detector positions, enabling calculation of expected dose values for comparison. All treatment plans generated in the TPS were subsequently exported to the delivery console, and the planned irradiations were executed on the HDR unit under controlled conditions.

### Comparison of calculated vs. measured dose

To evaluate the agreement between TPS-calculated and measured doses, the rectal probe was first positioned at Position 1, corresponding to a distance of 3.2 cm from the centre of the source transfer channel. The dwell position aligned with the rectal probe (R3) was activated for 300 s, and dose readings from detectors R1–R5 were recorded. For each detector position, the mean value of three consecutive measurements was calculated to quantify random variation. This procedure was then repeated with the detector placed at increasing distances of 4.2 cm, 5.2 cm, 6.2 cm, 7.2 cm, and 8.2 cm from the source to evaluate dose falloff and positional dependence. An identical methodology was used for the bladder probe, with measurements acquired by activating the dwell position corresponding to the centre of the bladder detector (B1).

### Linearity and reproducibility in measurements of detector

To evaluate the linearity of the diode detector response, the rectal probe was positioned at position 1, located 3.2 cm from the source transfer channel. The dwell position corresponding to the centre of the rectal detector (R3) was activated for an initial duration of 60 s, and dose readings from detectors R1–R5 were recorded. For each dwell time, the mean of three consecutive measurements was calculated to quantify random fluctuations. The procedure was then repeated for extended dwell times of 120, 180, 240, and 300 s, enabling assessment of detector response over a range of delivered doses. The same methodology was applied for the bladder probe, with measurements acquired by activating the dwell position aligned with the centre of the bladder detector (B1).

### Angular dependence

**Fig. 4:**
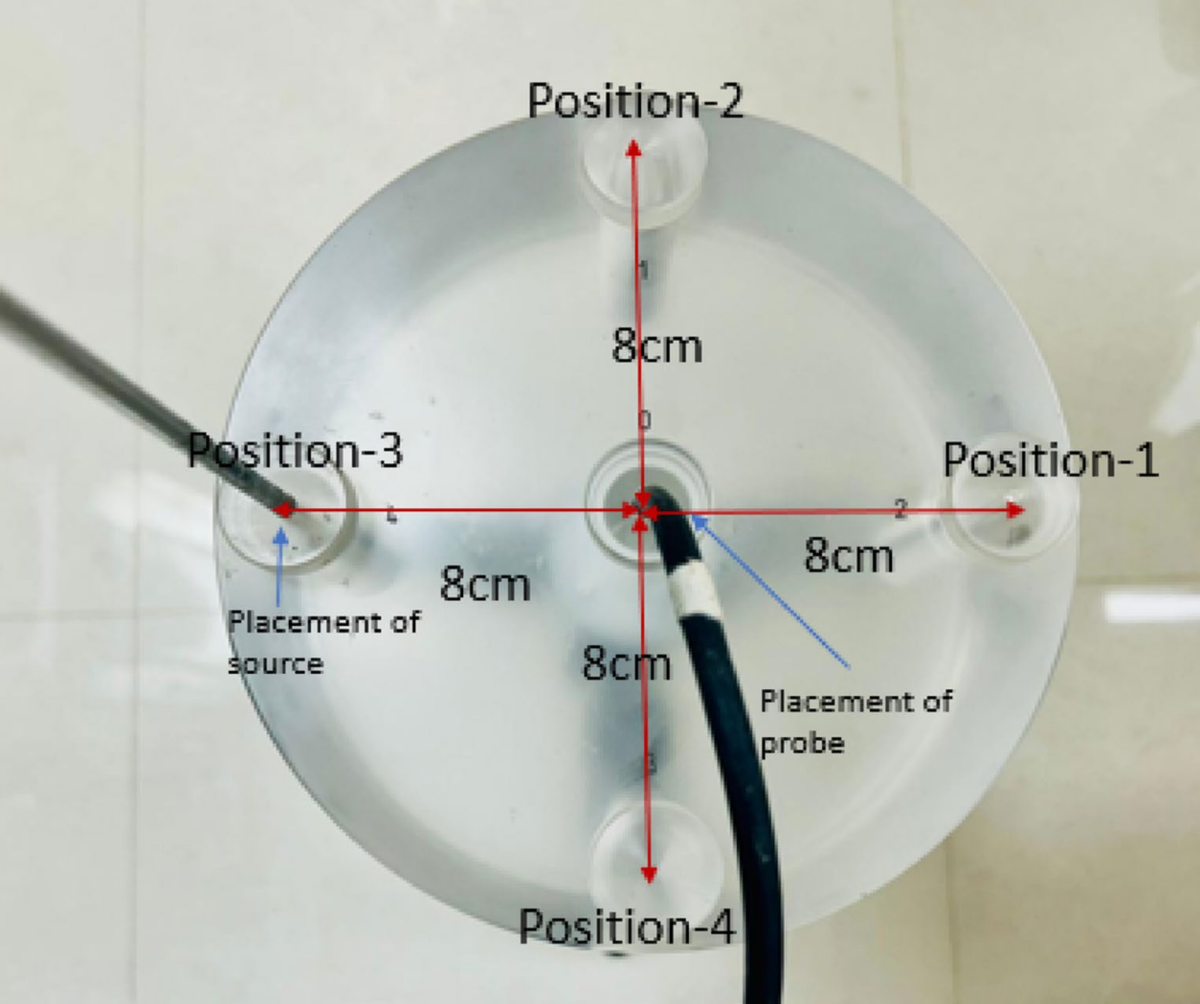
Four positions in calibration phantom used to measure angular dependence

To assess the angular dependence of the diode detector, measurements were performed using the dedicated calibration phantom, which contained five slots: one central slot and four peripheral slots (Position 1–4), each located 8 cm from the centre. Position 1 corresponded to 0°, Position 2 to 90°, Position 3 to 180°, and Position 4 to 270° relative to the detector orientation. The rectal probe was placed in the central slot, and the ^60^Co source was sequentially positioned in each of the peripheral slots. For each angular position, the dwell time was set to 60 seconds, and the corresponding detector readings were recorded. The mean of three consecutive measurements was calculated to quantify random variability. This procedure was repeated for all four peripheral positions to characterize the detector’s angular response. The same protocol was followed for the bladder probe, with the probe placed in the central slot and the ^60^Co source cycled through the peripheral slots (Fig. 4).

### Impact of tissue inhomogeneity

**Fig. 5:**
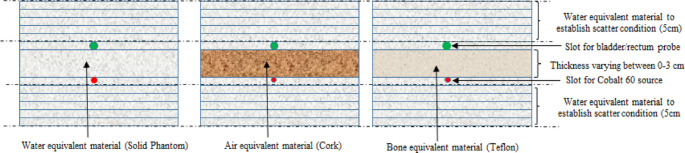
Experimental setup used to estimate any uncertainties in the presence of various inhomogeneous materials (water-equivalent, cork, Teflon)

To evaluate the impact of tissue inhomogeneity on detector accuracy, measurements were performed using materials of different densities that simulate clinically relevant tissue types—namely water-equivalent slabs, cork (lung/air equivalent), and Teflon (bone equivalent). The experimental setup consisted of a 5 cm water-equivalent layer to establish full scatter conditions, above which a custom adapter plate for the LLA 1400 universal transfer tube was positioned to guide the motion of the Co-60 source with fixed geometry. A corresponding adapter plate was used to position the rectal or bladder diode detector directly above the source channel, followed by another 5-cm water-equivalent layer placed superiorly to maintain symmetrical scatter conditions. Inhomogeneity materials of thickness 1.1 cm, 2.2 cm, and 3.3 cm were then inserted between the source and the detector to simulate progressive variations in tissue composition. The source was positioned between 5-cm water-equivalent plates to ensure full scatter conditions. The dwell position was activated such that it replicated the position of the R3 detector, thereby maintaining a fixed and clinically relevant source–detector geometry throughout the measurements. Figure 5 illustrates the experimental arrangement used to quantify measurement uncertainties in the presence of different inhomogeneity conditions.

## Results

### Comparison of calculated vs. measured doses

**Fig. 6:**
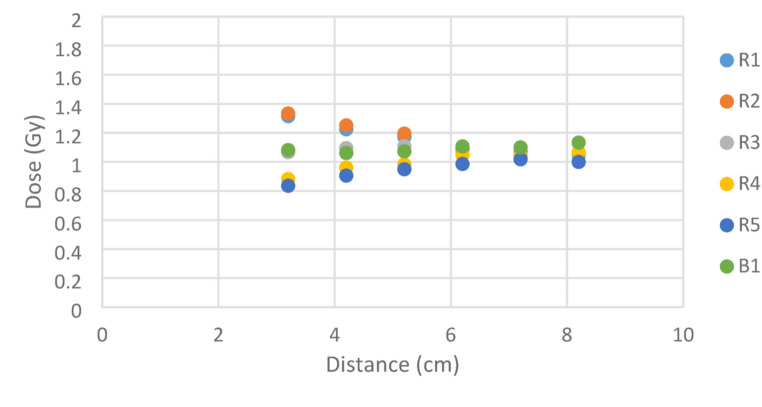
Comparison of TPS-calculated doses and the corresponding measured doses obtained using the PTW in-vivo diode detectors at various source–detector distances

Figure 6 compares TPS-calculated doses and the corresponding measured doses obtained using the PTW in-vivo diode detectors at various source detector distances. A noticeable trend is observed across the measurement positions: At distances closest to the source (e.g., 3.2–4.2 cm), detectors R1, R2, R4, and R5 show the largest discrepancies between measured and calculated values, with a maximum deviation of approximately 0.3 Gy. This increased variation near the source is expected due to the steep dose gradients that are characteristic of HDR brachytherapy, where even minimal positional differences or geometric uncertainties can result in substantial dose variation. In contrast, detectors R3 and B1, which were positioned within the same axial plane as the activated dwell position, exhibited minimal deviation from TPS-calculated doses. This highlights the importance of geometric alignment between detector and source dwell position, reducing angular and positional uncertainties in high-gradient regions. As the distance from the source increased beyond 5 cm, the discrepancies between calculated and measured values got smaller and became nearly negligible. This reduction reflects the gradual flattening of the dose gradient with increasing distance, resulting in a more uniform dose distribution and reduced sensitivity to small positional uncertainties.

### Linearity and reproducibility

**Fig. 7:**
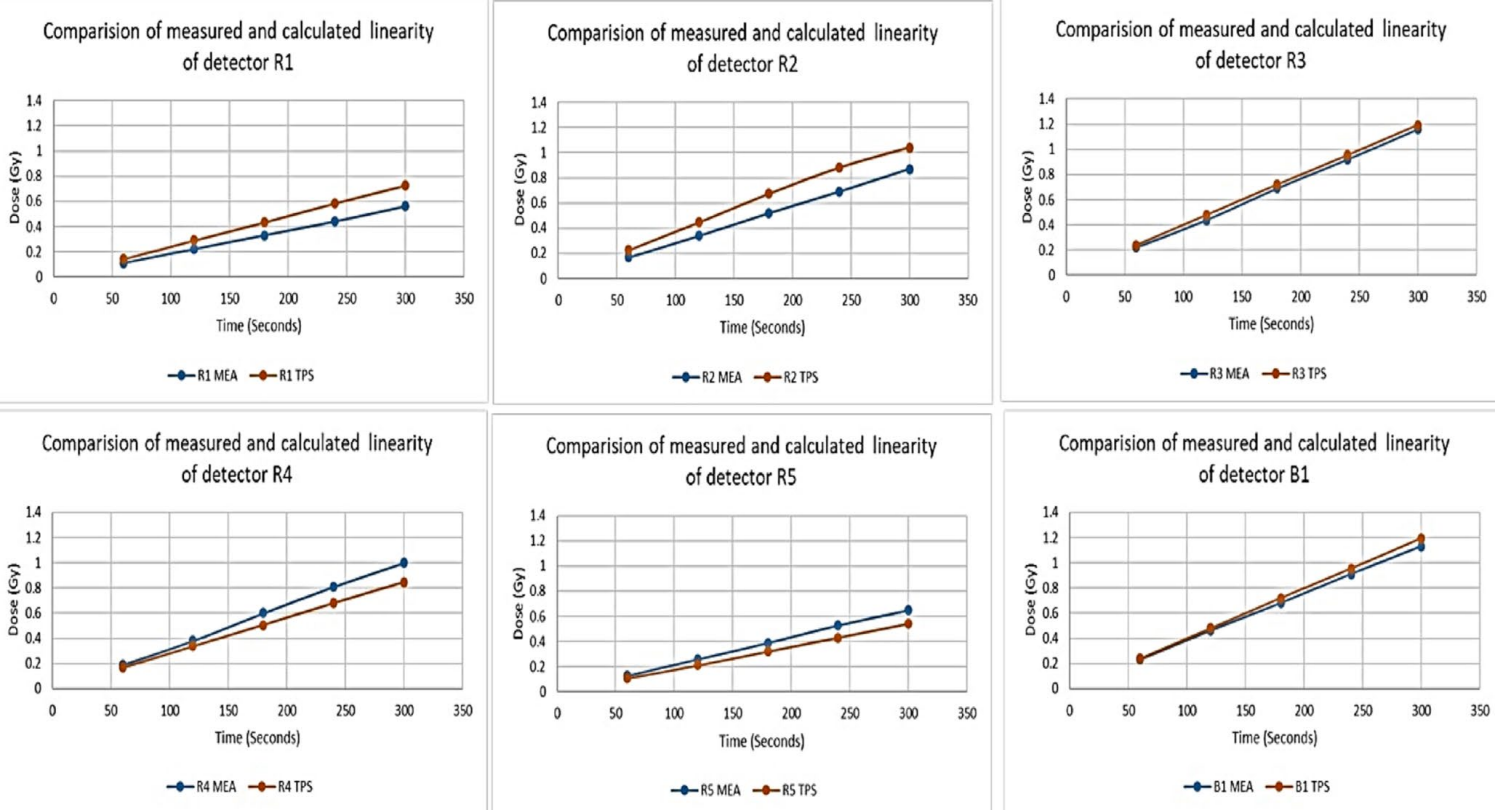
Doses measured (MEA; blue) and those calculated with the treatment planning system (TPS) (red) for diode detectors R1–R5 and B1 at different source–detector distances as a function of irradiation time for a ^60^Co high dose rate brachytherapy source

In Fig. 7, measured and calculated doses for detectors R1–R5 and B1 are compared in terms of dose linearity. All detectors demonstrated a strong linear dose–response relationship across the evaluated time intervals, confirming consistent detector performance. Among them, R3 and B1 showed the closest agreement between measured and TPS–calculated values. This close agreement can be attributed to their positioning in the same plane as the radiation source, resulting in minimal geometric or scattering-related deviations. Overall, the high degree of agreement between measurement and calculation across all detectors supports the accuracy and reliability of the system in quantifying dose delivery.

### Angular dependence

**Fig. 8:**
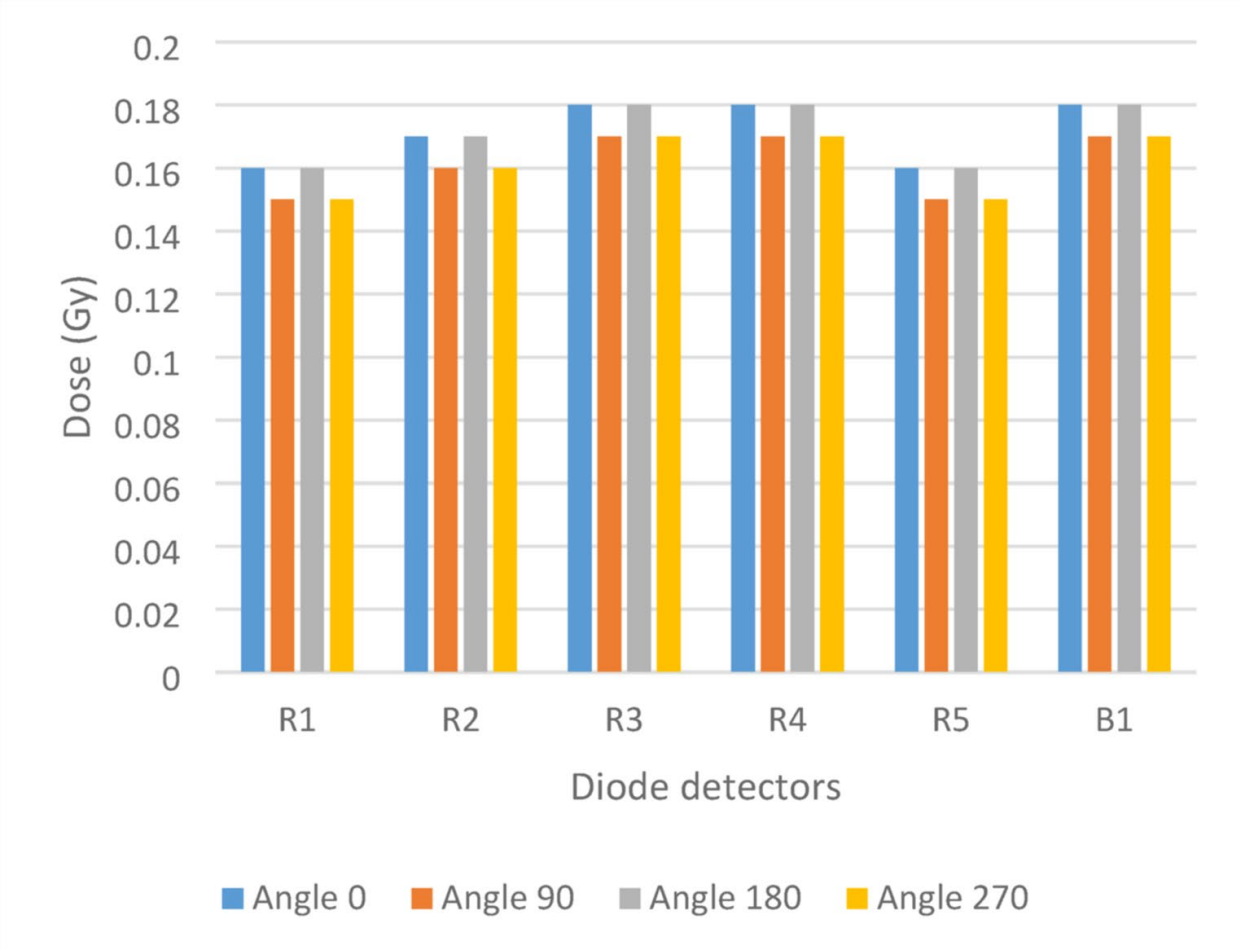
Angular dependence of PTW diode detectors at 0°, 90°, 180°, and 270° orientations. Involved uncertainties were less than 0.6%

Figure 8 depicts the angular dependence of the PTW diode detectors evaluated at 0°, 90°, 180°, and 270°. A measurable variation in detector response was observed with each 90° rotation; however, the magnitude of this variation remained small across all detectors (R1–R5 and B1). The observed small angular variation indicates that the detectors exhibit minimal angular sensitivity under the tested conditions, and the observed variations may be considered negligible for routine dosimetric use.

### Influence of material inhomogeneity

**Fig. 9:**
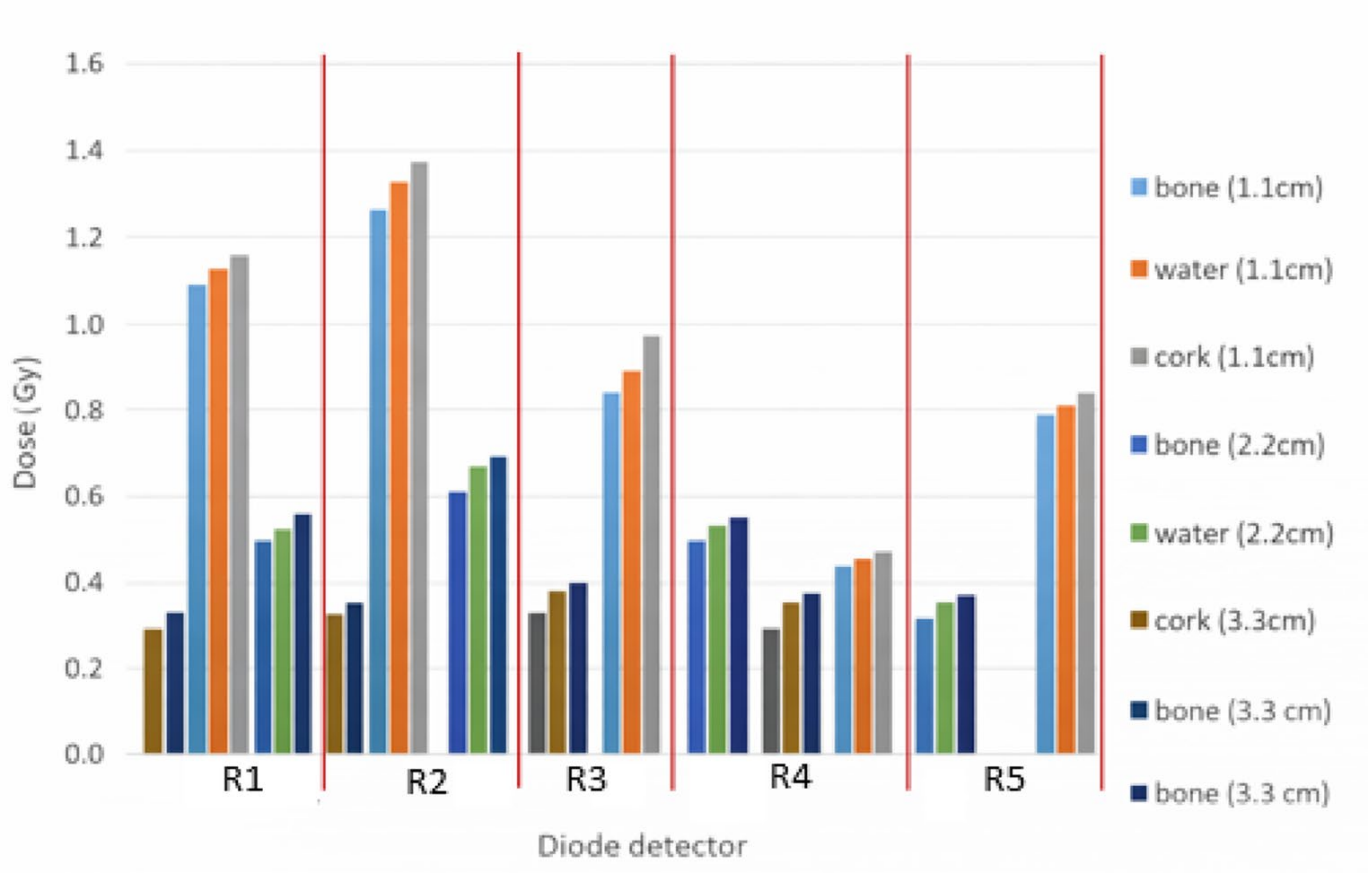
Detector response measured in the presence of inhomogeneous media of varying thicknesses (bone, water, and cork).Involved uncertainties were less than 0.4%

Figure 9 represents the detector response measured in the presence of different inhomogeneous media of varying thicknesses. When compared with 1.1 cm of water, a dose reduction of approximately 0.1 Gy was observed in the bone-equivalent medium, while the cork-equivalent medium produced an increased response of about 0.2 Gy. This trend persisted with increasing material thickness, indicating that even a 1 cm variation in medium composition can introduce a significant change in measured dose. These findings highlight the necessity of applying appropriate correction factors when performing dosimetry in the presence of inhomogeneities.

## Discussion

The present study evaluated the dosimetric performance of PTW in-vivo diode detectors in HDR brachytherapy and compared the observed doses with published reference doses. Near-source measurements (3–4 cm) showed dose deviations of 20–30%, which were considerably higher than the 5–10% reported by Tanderup et al. (2013). These elevated deviations likely reflect the extreme dose gradient and positional sensitivity inherent to semiconductor detectors positioned close to a HDR brachytherapy source. At larger distances (≥ 5 cm), deviations reduced to 5–8%, approaching the < 5% reported in low-gradient regions by Kertzscher et al. (2014) indicating that diode behaviour becomes more consistent where geometric sensitivity is reduced (Table 1).

**Table 1:** Comparison of percentage deviations observed in the present study with published literature; TPS – treatment planning system

Dosimetric Parameter	Current Study (Percentage Deviation)	Reported in Literature (Percentage Deviation)	Reference
TPS vs. measured dose deviation (near-source, 3–4 cm)	20–30% deviation (≈ 0.3 Gy over 1.0–1.2 Gy)	5–10% typical deviations in steep dose-gradient regions	Tanderup et al. (2013)
TPS vs. measured deviation at ≥ 5 cm distance	5–8%	< 5% in low-gradient regions	Kertzscher et al. (2014)
Angular dependence (0°, 90°, 180°, 270°)	10–15% variation	≤ 5–10% for most diode systems	Tanderup et al. (2013)
Effect of cork heterogeneity (1.1 cm thickness)	≈ 18% dose increase	10–20% increase	Faucher et al. (2025)
Material-thickness dependence (2.2–3.3 cm)	15–25% variation depending on density	10–25%	Faucher et al. (2025)
Overall system accuracy	Within AAPM TG-56 tolerance (5–10%) for most positions	Recommended clinical accuracy 5–10%	AAPM TG-56 (1997)
Clinical suitability (error sensitivity)	Angular + heterogeneity effects introduce 10–25% systematic influences	Error detection limited by geometry and medium effects	Kertzscher et al. (2014)

Heterogeneity corrections showed an 18% dose enhancement for cork, aligning with Faucher et al. (2025). Material-thickness effects between 15 and 25% also fell within the 10–25% range noted in prior phantom studies. These results support the need for localized, heterogeneity-specific correction factors for diode-based IVD.

The observed 14.7% dose reduction reported by Kwan et al. for ^192^Ir sources cannot be directly extrapolated to Co-60 HDR brachytherapy, as Co-60 emits higher-energy photons (1.17 and 1.33 MeV) compared with Ir-192 (average 0.35 keV to 0.38 keV), resulting in reduced photoelectric interaction, increased forward scatter, and lower sensitivity to low-density heterogeneities, which collectively mitigate dose perturbations under comparable geometric conditions.

Despite the deviations observed in the present study, the overall accuracy remained within the 5–10% tolerance recommended by AAPM TG-56 (Nath et al. 1997). However, combined angular and tissue heterogeneity-induced variations reaching 10–25% exceeded the geometry-driven limitations described by Kertzscher et (2014), suggesting that the PTW diodes in this configuration may be more susceptible to real-world perturbations.

In-vivo dosimetry has increasingly been adopted as a practical quality assurance tool in HDR brachytherapy, particularly for monitoring doses to organs at risk such as the rectum and bladder. Recent studies have shown that semiconductor diode–based systems are well suited for this purpose, as they provide real-time dose information with good reproducibility when appropriate calibration and geometric control are maintained (Carrara et al. 2018; Beaulieu et al 2011). Although much of the recent IVD literature focuses on ^192^Ir sources, the key performance characteristics of diode detectors—such as dose linearity, response stability, and reduced energy dependence at megavoltage photon energies—are also relevant to Co-60 HDR brachytherapy. These studies demonstrate that diode-based IVD systems can reliably detect deviations between planned and delivered dose in regions with steep dose gradients.

More recent reviews have highlighted that the higher photon energies of ^60^Co compared with ^192^Ir, result in reduced sensitivity to tissue heterogeneities and less pronounced energy-dependent detector response, which is advantageous for in-vivo dose verification (Fonseca & Beddar, 2020). At the same time, factors such as angular dependence and source–detector positioning remain important and must be considered, particularly in intracavitary brachytherapy applications (Chowdhury et al. 2024). The results of the present study are in line with these observations, showing stable diode response and good agreement between measured and TPS-calculated doses across multiple detector positions. This supports the clinical applicability of commercially available diode-based IVD systems for ^60^Co HDR brachytherapy as a reliable tool for routine quality assurance and patient safety. Highlights of the present study include demonstration of a reasonable dosimetric behaviour of PTW diodes under multiple geometric and material conditions that are typical for real-world clinical settings: a larger near-source and angular deviations than reported in existing literature, a confirmed reproducibility and linearity consistent with semiconductor dosimetry standards, and an overall compliance with AAPM TG-56 tolerances for most clinical conditions.

### Study Limitations

This study was conducted in a phantom environment, which may not fully replicate patient anatomy. Furthermore, only one diode system was evaluated, limiting cross-device generalization. Manual alignment may have contributed to elevated near-source deviations. Additionally, real-time clinical uncertainties such as catheter deformation and patient motion were not included, which may influence detector performance.

Although diode-based IVD is feasible and largely consistent with published performance standards, its successful clinical integration requires corrections for angular dependence, heterogeneity variations, and near-source sensitivity. Thus, adoption into routine brachytherapy workflows should include improved calibration models and automated error-detection frameworks.

## Conclusion

The present study demonstrated that PTW diode-based in-vivo dosimetry in HDR brachytherapy is reliable and consistent with published literature in most conditions. However, deviations in high-gradient and angular scenarios underscore the need for refined calibration and correction strategies. Future research should focus on multi-institutional validation and integration into comprehensive treatment-verification systems.

## Data Availability

No datasets were generated or analysed during the current study.

## References

[CR1] Beaulieu Therriault-Proulx F, Briere TM, Mourtada F, Aubin S, Beddar S, Beaulieu L (2011) A phantom study of an in vivo dosimetry system using plastic scintillation detectors for real-time verification of 192 Ir HDR brachytherapy. Med Phys 38:2542–2551 21776789 10.1118/1.3572229 PMC3104721

[CR2] Beddar AS, Briere TM, Mourtada FA, Vassiliev ON, Liu HH, Mohan R (2005) Monte Carlo calculations of the absorbed dose and energy dependence of plastic scintillators. Med Phys 32:1265–1269 15984678 10.1118/1.1897465

[CR3] Bergau PFL, Schirmer MA, Leha A, Leu M, Emons G, Hess CF, Hille A (2019) The impact of rectal/bladder filling and applicator positioning on in vivo rectal dosimetry in vaginal cuff brachytherapy using an enhanced therapy setting. Brachytherapy 19:168–175 31883803 10.1016/j.brachy.2019.11.009

[CR4] Carrara M, Cutajar D, Alnaghy S, Espinoza A, Romanyukha A, Presilla S, Tenconi C, Cerrotta A, Fallai C, Safavi-Naeini M, Petasecca M, Kejda A, Lerch M, Corde S, Jackson M, Howie A, Bucci J, Rosenfeld AB (2018) Semiconductor real-time quality assurance dosimetry in brachytherapy. Brachytherapy 17(1):133–145. 10.1016/j.brachy.2017.08.013 28964727 10.1016/j.brachy.2017.08.013

[CR5] Chowdhury AA, Bolton S, Lowe G, Vasquez Osorio E, Hamblyn W, Hoskin PJ (2024) The clinical application of in vivo dosimetry for gynaecological brachytherapy: A scoping review. Tech Innov Patient Support Radiat Oncol 33:100290 Published 2024 Nov 16. 10.1016/j.tipsro.2024.100290 39802319 10.1016/j.tipsro.2024.100290 PMC11718348

[CR6] Faucher J, Turgeon V, Bahoric B, Enger SA, Watson PGF (2025) Isolating the impact of tissue heterogeneities in high dose rate brachytherapy treatment of the breast. Phys imaging radiation Oncol 33:100737. 10.1016/j.phro.2025.100737 10.1016/j.phro.2025.100737 PMC11910349 40093657

[CR7] Fonseca GP, Johansen JG, Smith RL, Beaulieu L, Beddar S, Kertzscher G, Verhaegen F, Tanderup K (2020) In vivo dosimetry in brachytherapy: Requirements and future directions for research, development, and clinical practice. Phys imaging radiation Oncol 16:1–11. 10.1016/j.phro.2020.09.002 10.1016/j.phro.2020.09.002 PMC7807583 33458336

[CR8] Johan SK, Lobo D, Athiyamaan MS et al (2023) In-vivo Comparison of Planned and Measured Rectal Doses during Cobalt-60 HDR CT-based Intracavitary Brachytherapy Applications of Cervical Cancer Using the PTW 9112 Semiconductor Probe. Asian Pac J Cancer Prev 24(3):897–907 Published 2023 Mar 1. 10.31557/APJCP.2023.24.3.897 36974543 10.31557/APJCP.2023.24.3.897 PMC10334100

[CR9] Kertzscher G, Rosenfeld A, Beddar S, Tanderup K, Cygler JE (2014) In vivo dosimetry: trends and prospects for brachytherapy. Br J Radiol 87(1041):20140206 25007037 10.1259/bjr.20140206 PMC4453151

[CR10] Kwan IS, Wilkinson, Dd C, DIdi, Llerch M, Rosenfeld A (2009) The effect of rectal heterogeneity on waldo’s in hi to straight brachytherapy. Med phys 36(1):224–232 19235390 10.1118/1.3031111

[CR11] Mozaffari A, Ghorbani M (2019) Determination of TG-43 Dosimetric Parameters for Photon Emitting Brachytherapy Sources. *Journal of biomedical physics & engineering* vol. 9,4 425–436. 1 Aug. 10.31661/jbpe.v0i0.570 10.31661/jbpe.v0i0.570 PMC6709347 31531295

[CR12] Nath R, Anderson LL, Meli JA, Olch AJ, Stitt JA, Williamson JF (1997) Code of practice for brachytherapy physics: report of the AAPM Radiation Therapy Committee Task Group No. 56. American Association of Physicists in Medicine. Med Phys. ;24(10):1557-98. 10.1118/1.597966. PMID: 9350711 10.1118/1.597966 9350711

[CR13] Tanderup K, Beddar S, Andersen CE, Kertzscher G, Cygler JE (2013) In vivo dosimetry in brachytherapy. Med Phys 40(7):070902 23822403 10.1118/1.4810943

